# A Comprehensive Evaluation of Consensus Spectrum Generation Methods in Proteomics

**DOI:** 10.1021/acs.jproteome.2c00069

**Published:** 2022-05-13

**Authors:** Xiyang Luo, Wout Bittremieux, Johannes Griss, Eric W. Deutsch, Timo Sachsenberg, Lev I. Levitsky, Mark V. Ivanov, Julia A. Bubis, Ralf Gabriels, Henry Webel, Aniel Sanchez, Mingze Bai, Lukas Käll, Yasset Perez-Riverol

**Affiliations:** † Chongqing Key Laboratory of Big Data for Bio Intelligence, Chongqing University of Posts and Telecommunications, 400065 Chongqing, China; ‡ Skaggs School of Pharmacy and Pharmaceutical Sciences, University of California San Diego, La Jolla, California 92093, United States; § European Molecular Biology Laboratory, European Bioinformatics Institute (EMBL-EBI), Wellcome Genome Campus, Hinxton, Cambridgeshire CB10 1SD, U.K.; ∥ Department of Dermatology, Medical University of Vienna, 1090 Vienna, Austria; ⊥ Institute for Systems Biology (ISB), Seattle, Washington 98109, United States; # Applied Bioinformatics, Department for Computer Science, University of Tuebingen, Sand 14, 72076 Tuebingen, Germany; ∇ V.L. Talrose Institute for Energy Problems of Chemical Physics, N.N. Semenov Federal Research Center for Chemical Physics, Russian Academy of Sciences, Moscow 142432, Russia; ⊗ VIB-UGent Center for Medical Biotechnology, B-9052 Ghent, Belgium; ■ Department of Biomolecular Medicine, Ghent University, B-9000 Ghent, Belgium; □ Novo Nordisk Foundation Center for Protein Research, University of Copenhagen, Copenhagen DK-2200, Denmark; ● Section for Clinical Chemistry, Department of Translational Medicine, Lund University, Skåne University Hospital Malmö, 20502 Malmö, Sweden; ○ Science for Life Laboratory, School of Engineering Sciences in Chemistry, Biotechnology and Health, Royal Institute of Technology − KTH, Box 1031, 17121 Solna, Sweden

**Keywords:** mass spectrometry, clustering, spectral libraries, ProteomeXchange, big data, pride database, consensus spectra, benchmark

## Abstract

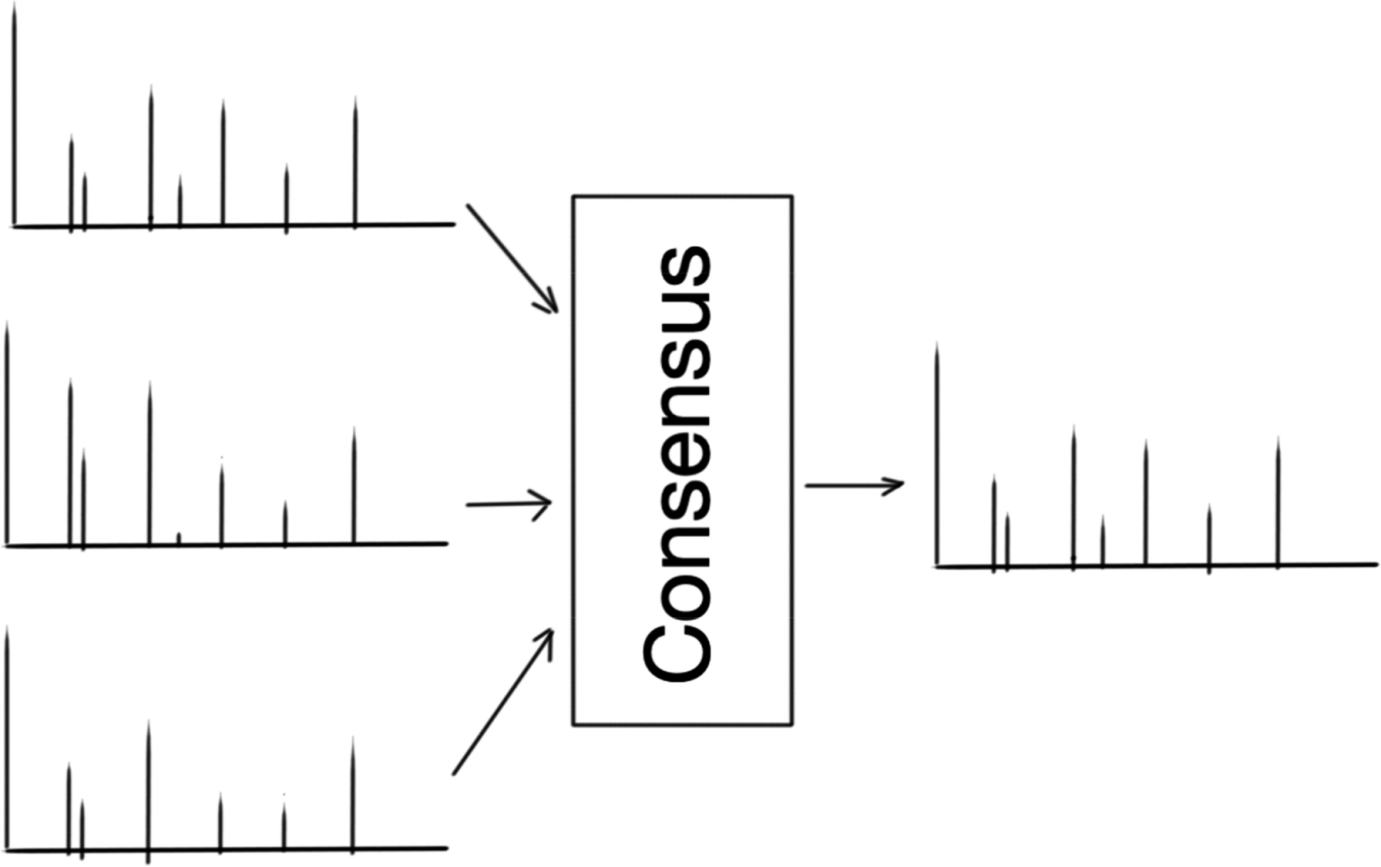

Spectrum clustering is a powerful strategy to minimize redundant mass spectra by grouping them based on similarity, with the aim of forming groups of mass spectra from the same repeatedly measured analytes. Each such group of near-identical spectra can be represented by its so-called consensus spectrum for downstream processing. Although several algorithms for spectrum clustering have been adequately benchmarked and tested, the influence of the consensus spectrum generation step is rarely evaluated. Here, we present an implementation and benchmark of common consensus spectrum algorithms, including spectrum averaging, spectrum binning, the most similar spectrum, and the best-identified spectrum. We have analyzed diverse public data sets using two different clustering algorithms (spectra-cluster and MaRaCluster) to evaluate how the consensus spectrum generation procedure influences downstream peptide identification. The BEST and BIN methods were found the most reliable methods for consensus spectrum generation, including for data sets with post-translational modifications (PTM) such as phosphorylation. All source code and data of the present study are freely available on GitHub at https://github.com/statisticalbiotechnology/representative-spectra-benchmark.

## Introduction

Spectrum clustering, i.e., the process of grouping similar spectra in a larger collection of MS2 spectra into smaller subsets, has multiple applications in mass spectrometry in general and in proteomics in particular,^1^ including the generation of spectral libraries^2^ and spectral archives,^3^ quality assessment of peptide identifications in public repositories, and improvement of quantification results.^4^ Spectrum clustering algorithms strive to group highly similar spectra so that each cluster contains spectra generated from the same analyte (peptidoforms with a specific charge in the case of proteomics). Differences between tools for spectrum clustering vary in their implementation of the various data processing steps, including the preprocessing of spectra (e.g., intensity normalization and peak picking), the clustering algorithm used, the metric used for determining similarity between spectra, and the optional optimizations to increase computational efficiency. Current tools for spectrum clustering include MS-Cluster,^3^ spectra-cluster,^2^ MaRaCluster,^5^ msCRUSH,^6^ and falcon.^7^

While the most apparent output of the process of spectrum clustering is a grouping of spectra into clusters, the majority of use cases benefit from a condensed single spectrum representation for each cluster. This is, for instance, useful for the data-driven creation of spectral libraries,^8^ for reannotation and visualization of clustering results in public data repositories,^2^ and for label-free quantification.^4^ The generation of high-quality representative spectra for each cluster is a key aspect of spectrum clustering, as the resulting consensus spectra form the starting point for downstream analyses. Although several spectrum clustering algorithms have been adequately benchmarked,^7,9^ the impact of the consensus spectrum generation procedure has so far not been properly evaluated. Several common approaches can be used to generate representative spectra, including spectrum binning, spectrum averaging,^3^ and selecting the most similar spectrum to all cluster members (medoid).^7^ Additionally, although this strategy can only be used for clusters that contain one or more identified spectra, the “best-identified spectrum” method uses the most confidently identified spectrum as cluster representative.^10^

Here, we have performed a comprehensive evaluation of algorithms for the generation of consensus spectra to assess their performance for downstream processing of spectrum clustering results. We have used the spectra-cluster and MaRaCluster tools to generate clusters from diverse publicly available data sets and explore whether consensus spectrum generation algorithms perform differently between different tools. Additionally, we have evaluated the impact of consensus spectrum generation on downstream peptide and protein identification performance. All code and analyses are open-source and available at https://github.com/statisticalbiotechnology/representative-spectra-benchmark under the permissive Apache 2.0 license.

## Methods

### Consensus Spectrum Generation Methods and Evaluation

For the benchmark, we implemented four consensus spectrum generation methods:

- Spectrum averaging (AVERAGE): The representative spectrum is an average of all the spectra in the cluster.^8,11,12^ In this algorithm, peaks with close *m*/*z* values are merged into a single peak, and their *m*/*z* values and intensities are averaged. *m*/*z* values are averaged using the corresponding peak intensities as weights.
- Spectrum binning (BIN): In this method, for each cluster, a consensus spectrum vector with a bin width of 0.02 *m*/*z* was first constructed.^12^ For all spectra in the cluster, peak *m*/*z* and intensity values were assigned to the corresponding bin in the consensus spectrum vector. Bins that contained values from fewer than 25% of the cluster members were discarded. Next, the vector was converted to a consensus spectrum by averaging all peak *m*/*z* and intensity values per bin.^2^
- Most similar spectrum (MOST): For each cluster, the spectrum that is on average most similar to all cluster members was selected as a representative spectrum.^13^ The most similar spectrum was selected by first calculating the dot product of all pairwise similarities between spectra in the cluster. Next, the spectrum with the maximal summed dot product to all other spectra was selected as the representative for that cluster.
- Best identified spectrum (BEST): For each cluster that contained at least one identified spectrum, the spectrum with the maximal peptide-spectrum match score was chosen as the representative for that cluster. Note that this approach is not valid if all spectra in the cluster are unmatched.

**Figure 1:**
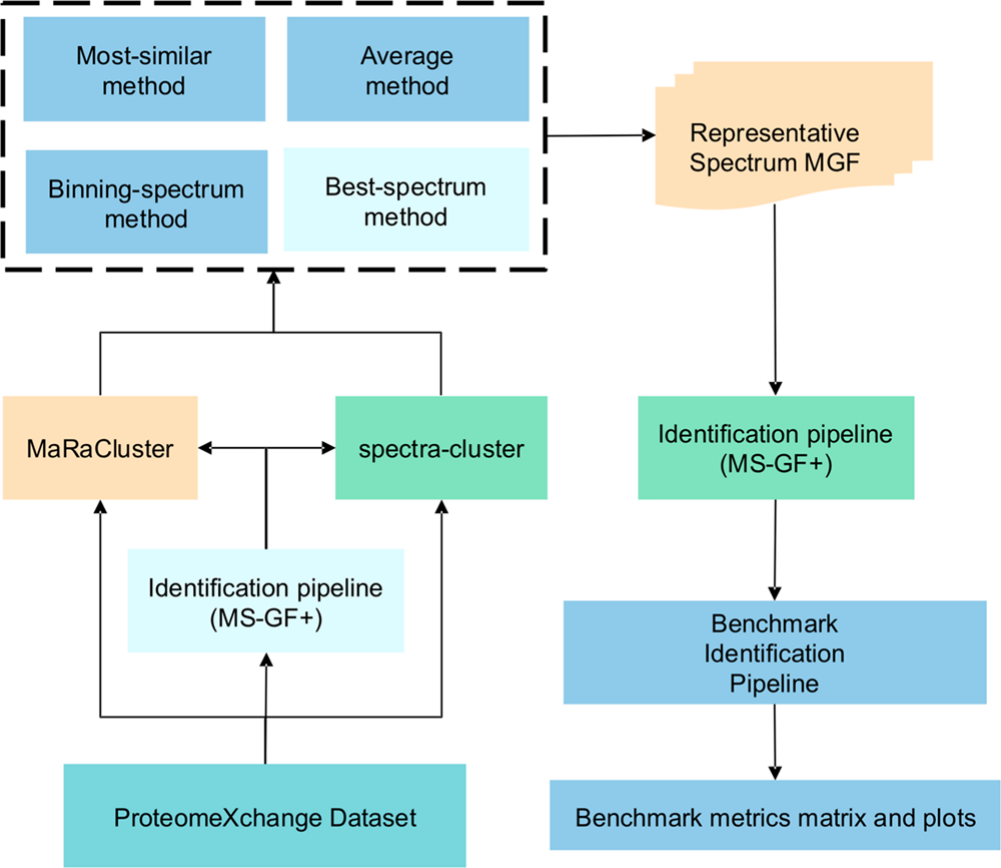
Study workflow, including clustering and peptide identification of publicly available ProteomeXchange data sets, consensus spectrum generation using alternative procedures, and evaluation of cluster representatives using an identification benchmark.

The data manipulation steps were implemented as reproducible Nextflow workflows (Figure 1). The spectra-cluster (version 1.1.2)^2^ and MaRaCluster (version 1.0)^5^ spectrum clustering tools were used to cluster the mass spectrum data, and the MS-GF+ sequence database search engine (version v2021.03.22)^14^ was used to perform peptide identification. For each cluster, representative (consensus) spectra were directly generated from the clustering output using the first three consensus generation procedures described above. For the best-identified method, the spectra were additionally identified using MS-GF+, after which the PSMs with the maximum scores were selected as representatives for each cluster. To ensure a fair comparison between all consensus spectrum generation procedures, clusters that only contained unidentified spectra were ignored, as no valid representative spectrum could be obtained using the best-identified method. To evaluate downstream peptide identification performance, the consensus spectra obtained for both spectrum clustering tools with each consensus spectrum generation method were searched using MS-GF+,^14^ after which the number of peptide identifications was compared between all combinations of clustering and consensus generation methods, and with the original data without clustering.

### Benchmark Datasets

We used four public ProteomeXchange data sets: PXD008355, PXD023047, PXD021518, and PXD023361 (Table 1). RAW data from each data set were converted to MGF using the ThermoRawFileParser (version: 1.2.3) tool^15^ with default parameters. Among them, PXD008355, PXD023047, and PXD021518 are from *Arabidopsis thaliana* (mouse-ear cress), and PXD023361 is from *Saccharomyces cerevisiae* (baker’s yeast). The data sets have been acquired using three different instrument models: Q Exactive, Q Exactive HF, and Q Exactive HF-X. The description of the samples, instrument configuration, sample processing steps, and analytical method can be read in the original publications: PXD008355,^16^ PXD023047,^17^ PXD021518,^18^ and PXD023361.^19^

**Table 1:** Datasets Were Reanalyzed to Evaluate the Performance of Each Consensus Spectrum Generation Algorithm^a^

project accession	instrument	no. MS/MS
PXD008355^16^	Q Exactive	1 477 567
PXD023047^17^	Q Exactive HF	109 333
PXD021518^18^	Q Exactive HF-X	286 410
PXD023361^19^	Q Exactive	38 286

a The number of peptide identifications and peptide-spectrum matches can be found in the Supplementary Notes. In addition, the description of each dataset can be found in the original publication and PRIDE Archive.^20^

For data sets PXD008355, PXD023047, and PXD021518, the *Arabidopsis thaliana* protein database was downloaded from http://ftp.ebi.ac.uk/pride-archive/2019/07/PXD008355/TAIR10.fasta, while for data set PXD023361 the *Saccharomyces cerevisiae* database was downloaded from http://ftp.pride.ebi.ac.uk/pride/data/archive/2021/04/PXD023361/uniprot-S_yeast.fasta.

For data sets PXD008355, PXD021518, and PXD023361, the precursor error tolerance was set to 10 ppm, while for data set PXD023047, it was set to 20 ppm. Target-decoy was performed using MS-GF+ (parameter -tda). For data sets PXD023047 and PXD021518 two modifications were allowed (NumMods = 2), fixed carbamidomethyl cysteine modification and variable methionine oxidation, while for data sets PXD008355 and PXD023361, phosphorylation was also considered as variable modification.

### Code Availability

All code and analyses are freely available as open source under the Apache 2.0 license at https://github.com/statisticalbiotechnology/representative-spectra-benchmark. The consensus generation procedures were implemented in Python 3.6. Software dependencies that were used include Matplotlib (version 3.1.2),^21^ Numba (version 0.47.0),^22^ NumPy (version 1.17.3),^23^ Pandas (version 0.25.3), pyOpenMS (version 2.4.0),^24^ Pyteomics (version 4.1.2),^25^ and spectrum_utils (version 0.3.3).^26^

## Results

### Impact of Consensus Clustering in Database Search Algorithms

**Figure 2:**
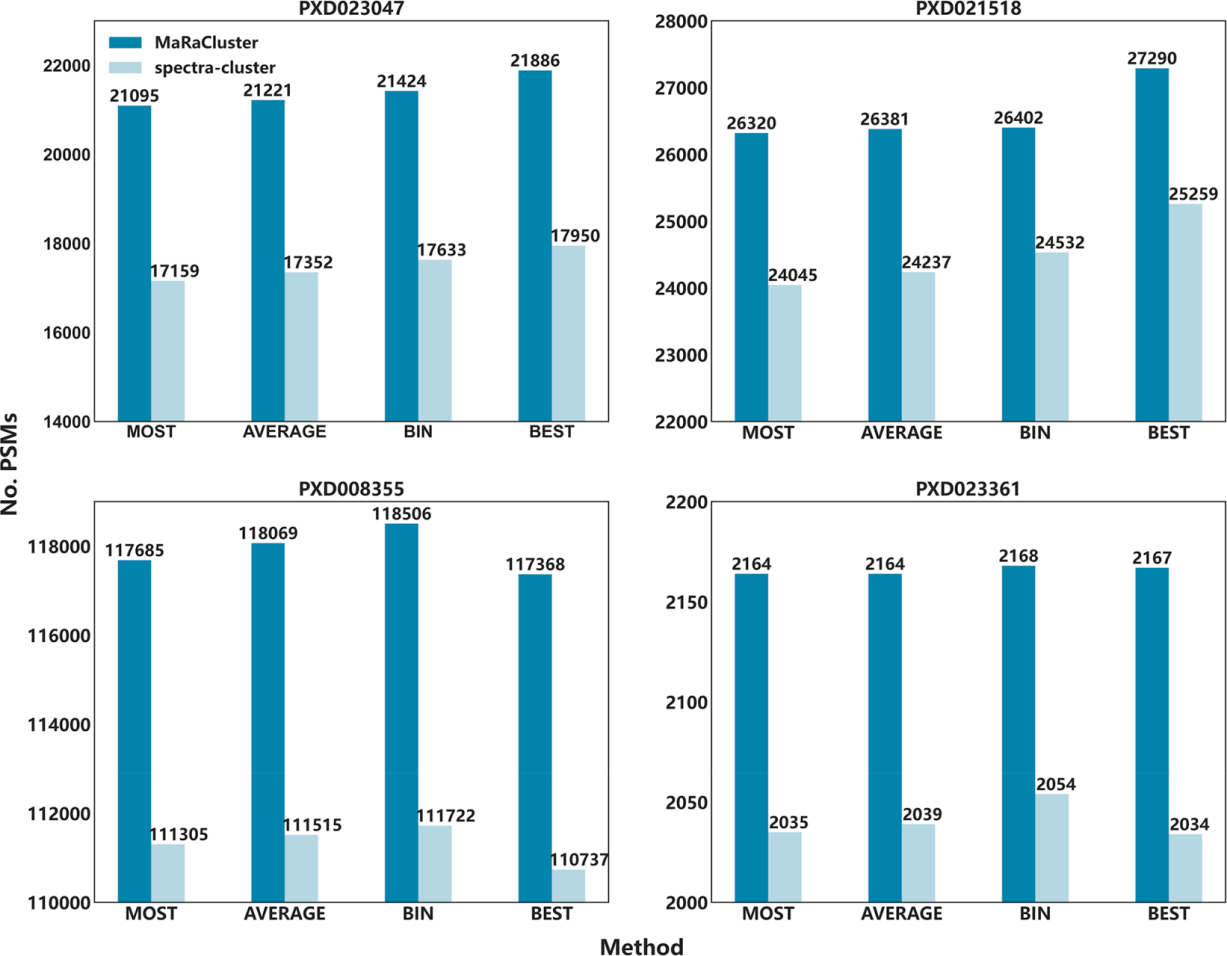
Number of PSMs obtained by MS-GF+ when searching consensus spectra produced by the MOST, AVERAGE, BIN, and BEST representative cluster generation methods for public proteomics data sets PXD023047, PXD021528, PXD008355, and PXD023361. Note that the bar plots are truncated past 0 to highlight relevant performance differences.

Figure 2 shows the number of PSMs (FDR = 1%) identified with MS-GF+ (data sets PXD023047, PXD021528, PXD008355, and PXD023361) for spectrum clustering using MaRaCluster and spectra-cluster followed by consensus spectrum generation using the MOST, AVERAGE, BIN, and BEST procedures. Among the four public proteomics data sets, whether using spectrum clustering results from MaRaCluster or spectra-cluster, the identification rate for the MOST method is lower compared to the other methods, while the BIN and BEST methods achieve a higher spectrum identification rate (Figure 2).

While the number of identified spectra only differs by a small amount between the various consensus spectrum generation procedures, when analyzing large public proteomics databases (billions of spectra)^27^ these differences can be translated into millions of spectrum identifications. Among the methods that transform the original spectra, the BIN method is the one that performed best. The BIN method divides the *m*/*z* range into bins and then integrates the intensities in the spectra that fall within those bins. The method favors the most intensive peaks, which might be a reason for the improved identification rates.

**Figure 3:**
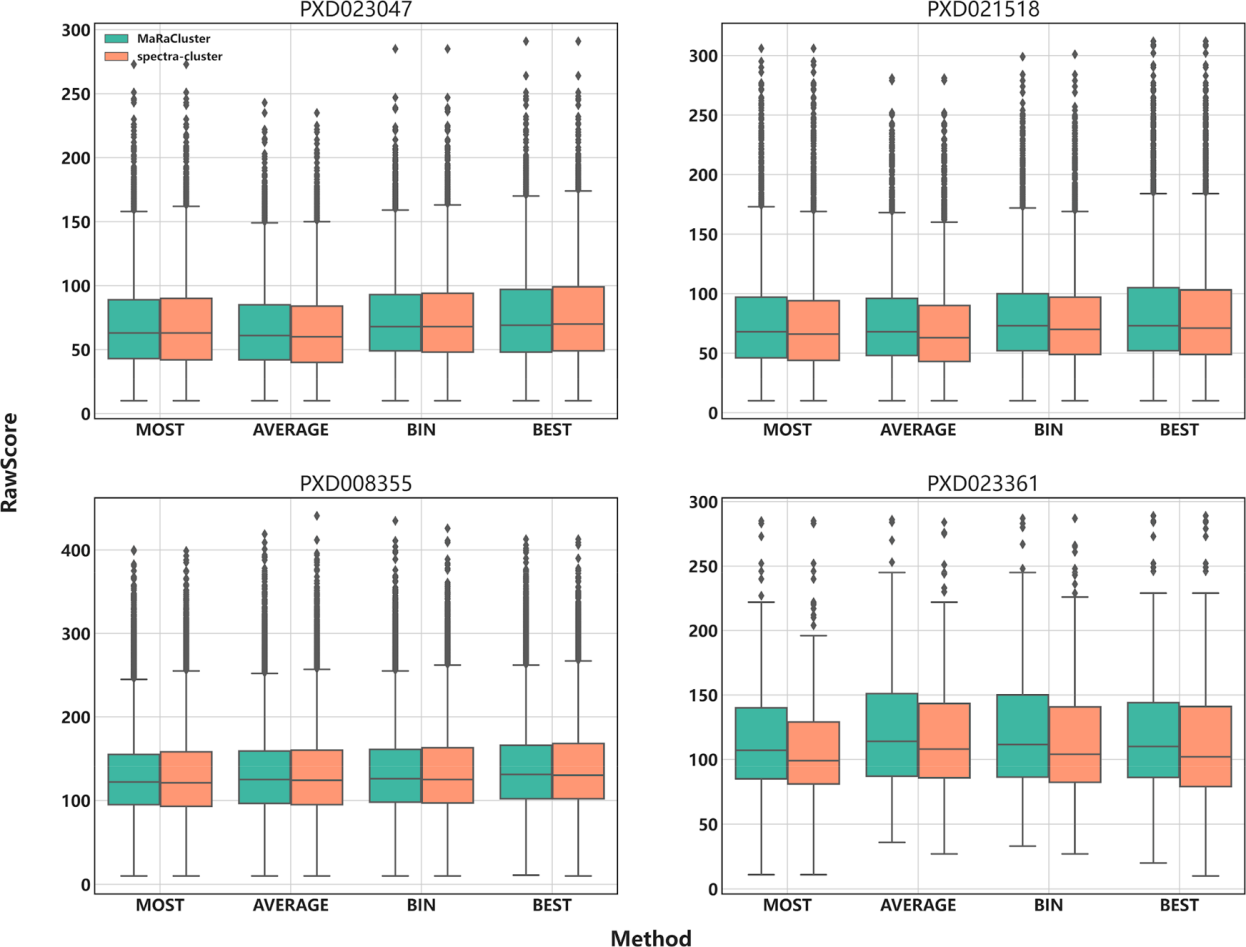
Distribution of MS-GF+ RawScores for MOST, AVERAGE, BIN, and BEST representative spectra from the public proteomics data sets PXD023047, PXD021528, PXD008355, and PXD023361.

Most of the consensus generation methods modify the original spectra, not only by removing or keeping some of the spectrum peaks but also by modifying the corresponding intensity of each peak. We have used the distributions of the MS-GF+ RawScore to explore the relationship between the final spectra and the quality of the peptide identifications. Figure 3 shows the distribution of MS-GF+ RawScore for the four consensus generation methods (MOST, AVERAGE, BIN, and BEST) after clustering with MaRaCluster and spectra-cluster. For both clustering tools, the BIN and BEST methods generate consensus spectra with higher average RawScore values (Figure 3), and similar to the previous metric (number of PSMs), the BEST algorithm achieves the highest average RawScore (Supplementary Note S1). The representative consensus spectra generated by the MOST method have the lowest average RawScore (Figure 3). The distribution of RawScore values (Figure 3) shows that the RawScores are more homogeneous for the BIN method (lower standard deviation) than for all the other methods, including the BEST algorithm (Supplementary Note S1).

**Figure 4:**
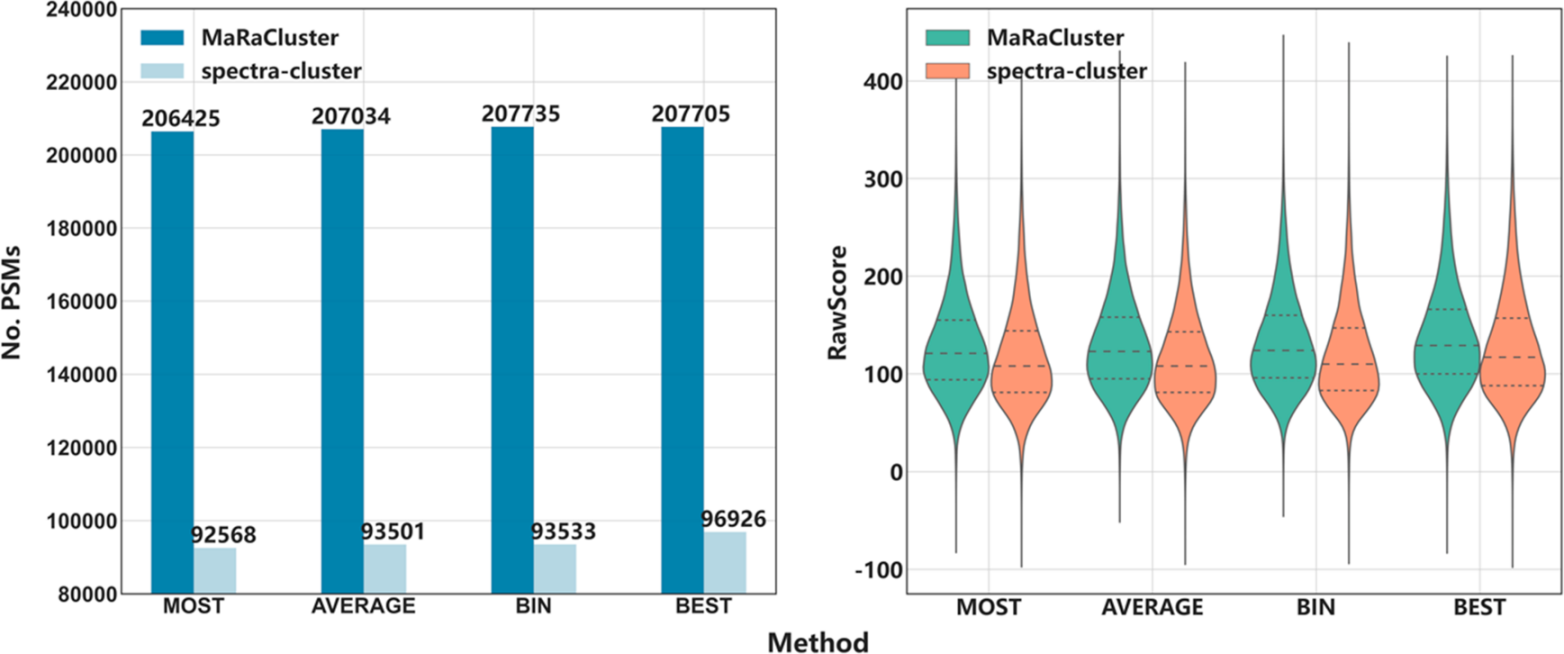
Number of PSMs and distribution of MS-GF+ RawScores for MOST, AVERAGE, BIN, and BEST representative spectra from the combined spectrum data (PXD023047, PXD021528, PXD008355, and PXD023361). Note that the bar plots are truncated past 0 to highlight relevant performance differences.

Furthermore, we compare the clustering algorithms and consensus methods for all the spectra of the four data sets combined (PXD023047, PXD021528, PXD008355, and PXD023361). Similar to the analysis performed in individual data sets, when the data was combined, the MOST and AVERAGE performed worse than BEST and BIN. This experiment shows that the instrument has little influence on the four representative spectrum generation methods (Figure 4).

The MOST method calculates the similarity distance between each spectrum in the cluster and other spectra in the cluster, sums the similarity distances, and finally selects the spectrum with the largest sum of similarity distances as the representative spectrum. For cases where the sum of multiple distances is the same, MOST always randomly selects a spectrum to represent. This process is likely to make the representative spectrum selected by MOST not truly representative of the entire cluster. And the selected representative spectrum is likely to be suboptimal (or even the worst) in the MS-GF reference score, which may also cause the representative spectrum to be screened out in the quality control process.

### Impact of Cluster Size and Quality of Peptide Identifications

**Figure 5:**
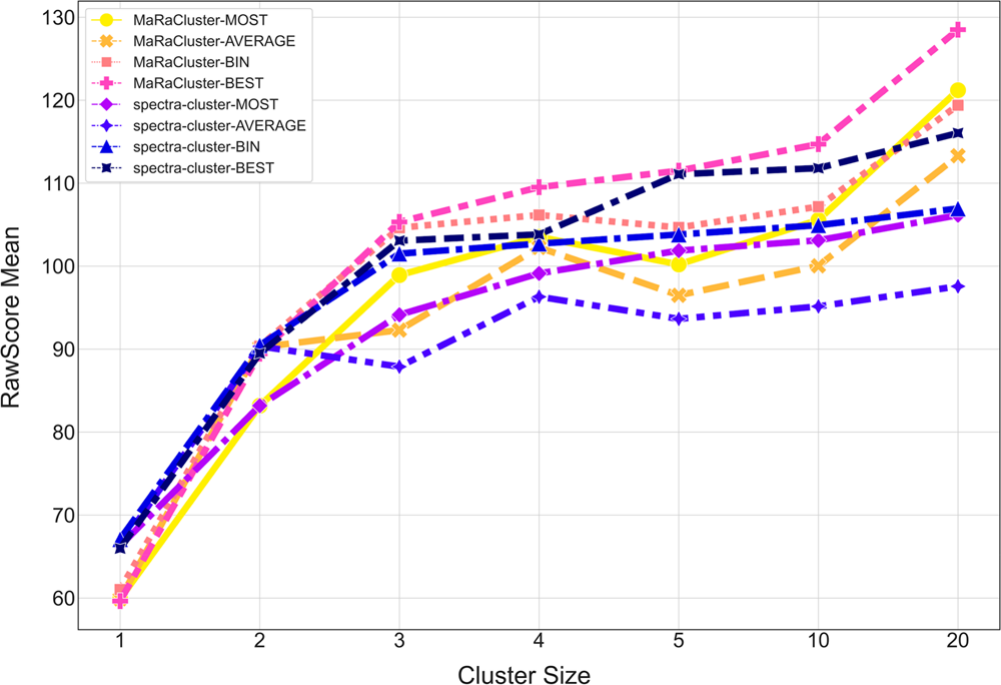
Average RawScore for the evaluated methods as a function of cluster size (1, 2, 3, 4, 5, 5–10, 10–20, 20 or higher).

Figure 5 shows the changes in mean RawScore of the identified spectra generated with the four evaluated methods (MOST, AVERAGE, BIN, and BEST) for clusters of different sizes (cluster sizes 1, 2, 3, 4, 5, 5–10, 10–20, 20 or higher). As expected, for clusters of a single spectrum, no differences were observed between different consensus methods, but minor differences were observed between the clustering algorithms. For other small clusters containing three or fewer spectra, consensus spectra derived from the spectra-cluster results, in combinations with all the consensus spectrum generation methods, provide higher mean RawScores than consensus spectra derived from MaRaCluster results. In contrast, for larger clusters, MaRaCluster consensus spectra lead to higher mean RawScores. For both spectra-cluster and MaRaCluster, the mean RawScore increases with increasing cluster size. The BEST and BIN algorithms are stable for both clustering algorithms and all data sets (Supplementary Note S1), and the scores of these two algorithms are generally higher than MOST and AVERAGE. In combination with MaRaCluster, the AVERAGE algorithm shows instability and the score of the AVERAGE algorithm is generally lower than the other three algorithms.

In addition, we explored how the clustering quality and consensus spectrum generation can impact the accuracy of the identification results. For example, which analyte will be identified from the consensus spectrum if a cluster contains four spectra and three were generated from the same analyte, but the last spectrum was generated from a different one? Will the search engine identify the most predominant or the divergent peptide?

We used the data of the four experiments combined to benchmark the quality of peptide identifications from the multispectral clusters, i.e., clusters that contained more than one spectrum. We hence removed singleton clusters, as well as clusters where there was no single peptide that appeared in more than 50% of the peptide-spectrum matches to its constituent spectra. We subsequently divided our clusters into two categories based on the relations between their representative spectra and their corresponding clustered spectra, the case when the consensus spectrum matched (1) the same or (2) a different peptide than the majority of the spectra in the cluster. We refer to these cases as high- and low-quality representative spectra.

Table 2 summarizes the results for the comparison of the matches to the spectra in the multispectral clusters and their representative spectrum. MaRaCluster showed better performance for all the consensus methods compared with spectra-cluster. For both clustering algorithms, the BEST method presented the best ratio of high-quality identified representative spectra, followed by the BIN, MOST, and/or AVERAGE methods. For spectra-cluster, which showed a higher number of mixed clusters, the number of high-quality representative spectra was higher for the BIN methods than for BEST. That difference is mainly due to the differences in the number of available clusters, after removing the clusters where no single peptide appeared in more than 50% of the peptide-spectrum matches, there were more multispectral clusters for the BEST than the BIN method (103 955 vs 102 639 clusters), but the methods have approximately the same number of high-quality representative spectra (80 577 vs 80 441).

**Table 2:** Fraction of the High- and Low-Quality Representative Spectra from Multispectral Clusters

methods	high-quality representative spectrum ratio	low-quality representative spectrum ratio
MaRaCluster BEST	0.871	0.129
MaRaCluster BIN	0.853	0.135
MaRaCluster AVERAGE	0.831	0.143
MaRaCluster MOST	0.842	0.158
spectra-cluster BEST	0.776	0.224
spectra-cluster BIN	0.779	0.212
spectra-cluster AVERAGE	0.770	0.215
spectra-cluster MOST	0.772	0.228

Due to the characteristics of the BIN and AVERAGE methods, some of the representative spectra have different peptides from all spectra in their corresponding multispectral clusters, which does not occur in BEST and MOST. The vast majority of poor representative spectra arise from situations where the peptides identified by the representative spectra are the same as those identified by a small fraction of the spectra in the cluster. The statistical results indicate that the representative spectra corresponding to the BEST and BIN methods are of higher quality, and the results of the representative spectra generation methods are more stable.

### Consensus Spectrum Generation Methods for Spectra Library Search

**Figure 6:**
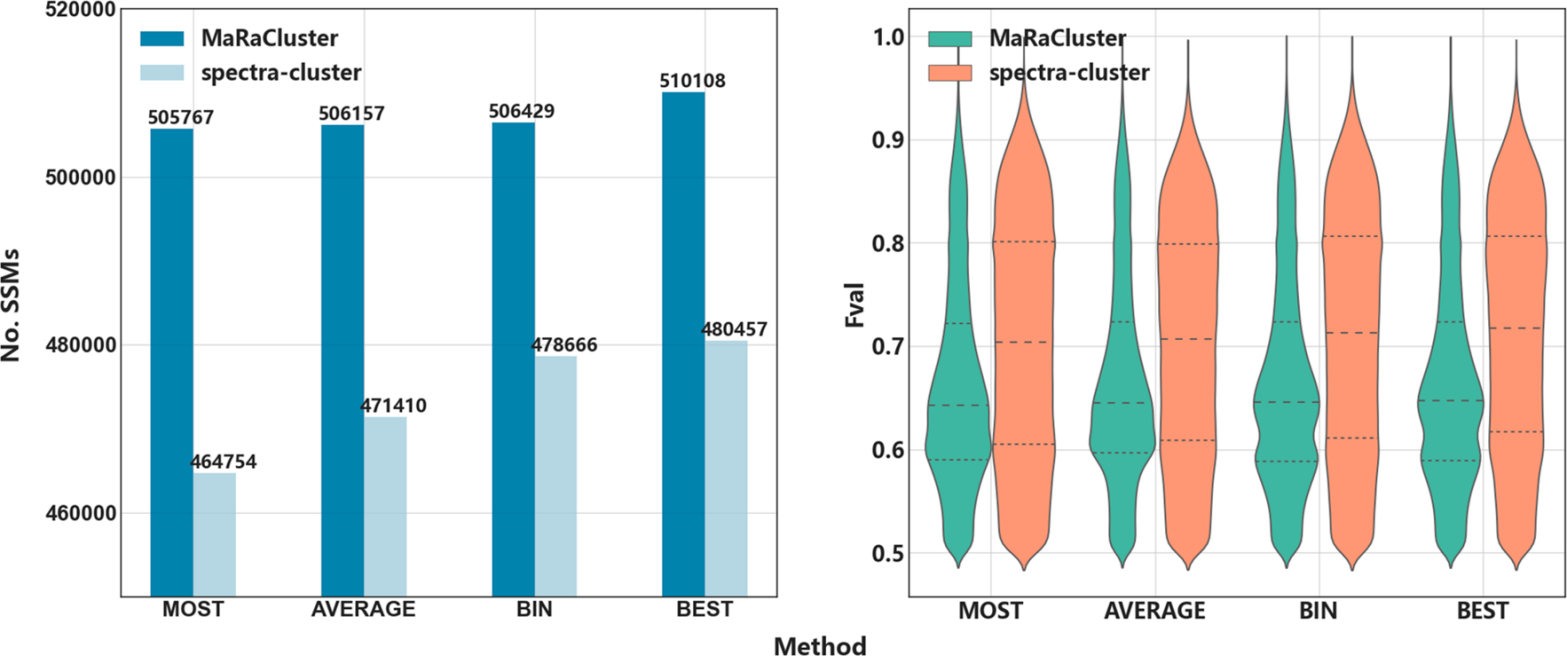
Number of spectral-spectral matches (SSMs) and distribution of Fval (SpectraST identification score) for MOST, AVERAGE, BIN, and BEST representative spectra from the combined spectrum data (PXD023047, PXD021528, PXD008355, and PXD023361).

We studied the impact of peptide identification for spectral library search approaches using the four different methods to create spectral libraries. In spectral library searches, all the consensus spectra are identified peptides. We performed a spectral library search on the combined data of the four methods using SpectraST (version: 5.0). We use the four methods to generate consensus spectra of BEST, BIN, MOST, and AVERAGE derived from the MS-GF+ matches for both clustering tools (MaRaCluster and spectrum-cluster), resulting in 8 spectral libraries in total). We used a threshold of the SpectraST scores, Fval ≥0.5, as the quality control criterion to perform statistical analysis on the postcharge search results (Figure 6).

In terms of the number of identifications, the results of spectral searches using SpectraST repeated the pattern of the previous experiments. The lowest number of matches was obtained against the library generated with the MOST method, followed by AVERAGE and BIN methods, and the BEST libraries performed better than the others. We explored the Fval (identification score) distribution of the identified peptides with each combination (Figure 6). No major differences are observed across consensus spectra generation; however, the Fval values are significantly higher when using spectra-cluster for clustering compared with MaRaCluster.

### Posttranslational Modification Site Localization of Consensus Spectra

In addition to peptide identification, we explored how using consensus spectra instead of the original spectra affects phospho-peptide identification and phosphorylation site localization. We analyzed the number of phosphorylation sites identified in data set PXD008355 after clustering with both tools (MaRaCluster and spectra-cluster) and the four different consensus spectrum generation methods (MOST, AVERAGE, BIN, and BEST). We have evaluated two metrics, (i) the number of phosphorylated PSMs identified and (ii) the phosphorylation sites identified.

**Figure 7:**
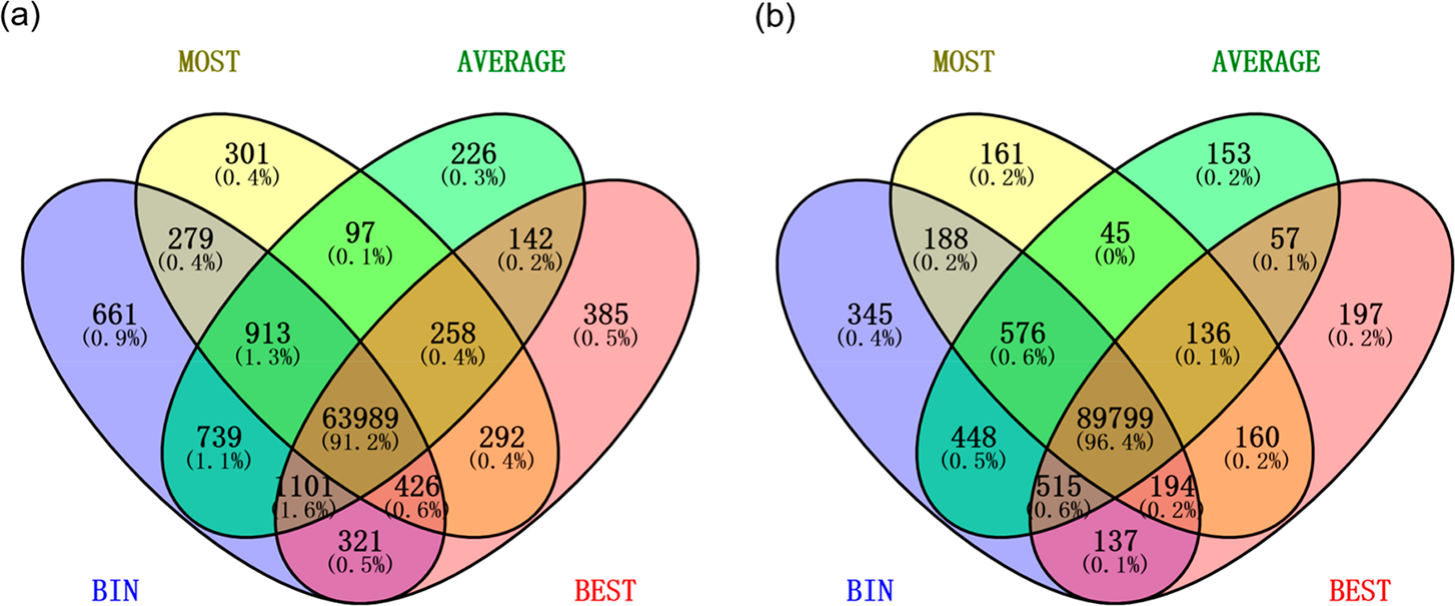
Intersection of the total phosphorylated PSMs among the four representative cluster methods in (a) MaRaCluster and (b) spectra-cluster.

Figure 6 shows the intersection of the phosphorylated PSMs among the four representative cluster methods after spectrum clustering with MaRaCluster and spectra-cluster. Most of the PSMs (91.2% for MaRaCluster and 96.4% for spectra-cluster) for the four representative cluster methods produce the same phosphorylated PSMs. The BIN method produces the largest number of unique PSMs, which is about double the number of other methods, followed by the BEST, MOST, and AVERAGE methods (Figure 7).

While the majority of phosphorylated PSMs are aggregated among all methods, around ∼1% are different and we also observed differences in terms of phosphorylation sites. Table 3 shows the difference in phosphorylation sites between BIN and BEST representative spectra from MaRaCluster and spectra-cluster (extended table, Supplementary Note S3). Because the BEST and BIN methods were the best performing consensus generation^12^ options in terms of peptide identification, we focus the discussion on these two methods (extended table, Supplementary Note S3). Most phosphorylated PSMs (63 165 for MaRaCluster and 89 161 for spectra-cluster) have their spectra mapped to peptides and phosphorylation sites in common for the BEST and BIN methods. We here call such PSMs corroborative. However, for a small number of PSMs, there were differences in the mapped phosphosites between the consensus spectrum generation methods (2683 in MaRaCluster and 1494 in spectra-cluster), which we here refer to as divergent PSMs. These small differences can be attributed to the fact that the BIN method modifies the ion peak intensity and *m*/*z* of the spectrum through the binning algorithm.

**Table 3:** Analysis of Phosphorylation Sites Identification of Dataset PXD008355, after Clustering with MaRaCluster and Spectra-Cluster, and Generation of the Consensus Spectra Using Two Different Methods (BEST, BIN)^a^

cluster method	method	phospho PSMs	phosphosites	corroborative PSMs	divergent PSMs
MaRaCluster	BEST	66 914	81 238	63 165	2683
	BIN	68 429	83 091		
spectra-cluster	BEST	91 195	109 877	89 161	1494
	BIN	92 202	111 230		

a We quantified the number of total phosphorylated PSMs and phosphorylation sites for each combination of clustering method and consensus generation method. In addition, we added the number of PSMs that ended up being in common or different from each cluster (corroborative and divergent PSMs) when comparing PSMs for the BEST and BIN method’s spectra.

## Conclusions

Representative spectra from clusters have typically been generated using four different algorithms: spectrum averaging, spectrum binning, the most similar spectrum, and the best-identified spectrum. Most tools and resources, including SpectraST,^8^ MassIVE^28^ spectral libraries, or spectra-cluster and PRIDE Cluster^2^ use one of these methods. However, to our knowledge, no systematic analysis has been performed to compare multiple algorithms to generate consensus spectra. We implemented a Python framework to benchmark existing algorithms to generate representative spectra from clustering results from two different popular clustering tools—MaRaCluster and spectra-cluster.

The BEST and BIN methods were found to be the most reliable methods for consensus spectrum generation, including for data sets with post-translational modifications such as phosphorylation. The BEST method generates representative consensus spectra based on existing spectrum identification results, which requires that all clusters contain identified spectra. Therefore, the BEST method cannot be used on spectral archives (clusters of nonidentified spectra) or if clustering is performed before the identification step. The BIN method is based on the original spectrum file and binning algorithm to generate representative consensus spectra and performed best in all benchmarks and comparisons after the BEST method. While the BIN algorithm modifies the original spectra, we do not observe major differences in identifying phosphorylated peptides and phosphorylation sites compared to the results of the BEST method to generate representative spectra. The fact that the BEST method is performing so well, compared to existing methods, suggests that better algorithms could be developed in the future to generate consensus spectra from clustering results.

## Supporting Information Available

Supplementary Note S1: Identification score as a function of cluster size; Supplementary Note S2: The data sets used in the benchmark; Supplementary Note S3: Analysis of the phosphoproteomics data set PXD008355 (PDF)
