# Supplementary material for: A Comprehensive Evaluation of Consensus Spectrum Generation Methods in Proteomics
Source: J Proteome Res. 2022 May 13;21(6):1566–74. doi: 10.1021/acs.jproteome.2c00069 (PMC9171829; doi:10.1021/acs.jproteome.2c00069)
Supplement: Supplementary file 1 — pr2c00069_si_001.pdf [file pr2c00069_si_001.pdf]

A comprehensive evaluation of consensus spectrum generation methods in proteomics

Xiyang Luo [1, &], Wout Bittremieux [2, &], Johannes Griss [3,4], Eric W Deutsch [5], Timo Sachsenberg [6], Lev I. Levitsky [7], Mark V. Ivanov [7], Julia A. Bubis [7], Ralf Gabriels [8,9], Henry Webel [10], Aniel Sanchez [11], Mingze Bai [1], Lukas Käll [12, *] and Yasset Perez-Riverol [3,*]

[1] Chongqing Key Laboratory of Big Data for Bio Intelligence, Chongqing University of Posts and Telecommunications, 400065, Chongqing, China.

[2] Skaggs School of Pharmacy and Pharmaceutical Sciences, University of California San Diego, La Jolla, California, 92093, United States.

[3] European Molecular Biology Laboratory, European Bioinformatics Institute (EMBL-EBI), Wellcome Genome Campus, Hinxton, Cambridgeshire, CB10 1SD, UK.

[4] Department of Dermatology, Medical University of Vienna, 1090 Vienna, Austria.

[5] Institute for Systems Biology (ISB), Seattle, Washington 98109, USA.

[6] Applied Bioinformatics, Department for Computer Science, University of Tuebingen, Sand 14, 72076 Tuebingen, Germany.

[7] V.L. Talrose Institute for Energy Problems of Chemical Physics, N.N. Semenov Federal Research Center for Chemical Physics, Russian Academy of Sciences, Moscow, 142432, Russia

[8] VIB-UGent Center for Medical Biotechnology, 9052, Ghent, Belgium

[9] Department of Biomolecular Medicine, B-9000, Ghent University, Belgium

[10] Novo Nordisk Foundation Center for Protein Research, University of Copenhagen, DK-2200, Denmark

[11] Section for Clinical Chemistry, Department of Translational Medicine, Lund University, Skåne University Hospital Malmö, 205 02 Malmö, Sweden

[12] Science for Life Laboratory, School of Engineering Sciences in Chemistry, Biotechnology and Health, Royal Institute of Technology - KTH, Box 1031, 17121 Solna, Sweden.

[&] Xiyang Luo and Wout Bittremieux: These authors contributed equally to this work. * Corresponding authors Yasset Perez-Riverol (yperez@ebi.ac.uk) and Lukas Käll (lukas.kall@scilifelab.se).

Supplementary Notes

Table of Contents

Supplementary Note 1: Identification score as a function of cluster size 3

Supplementary Note 2: The benchmark datasets 5

Supplementary Note 3: Analysis of the phosphoproteomics dataset. 6

Supplementary Note 1: Identification score as a function of cluster size

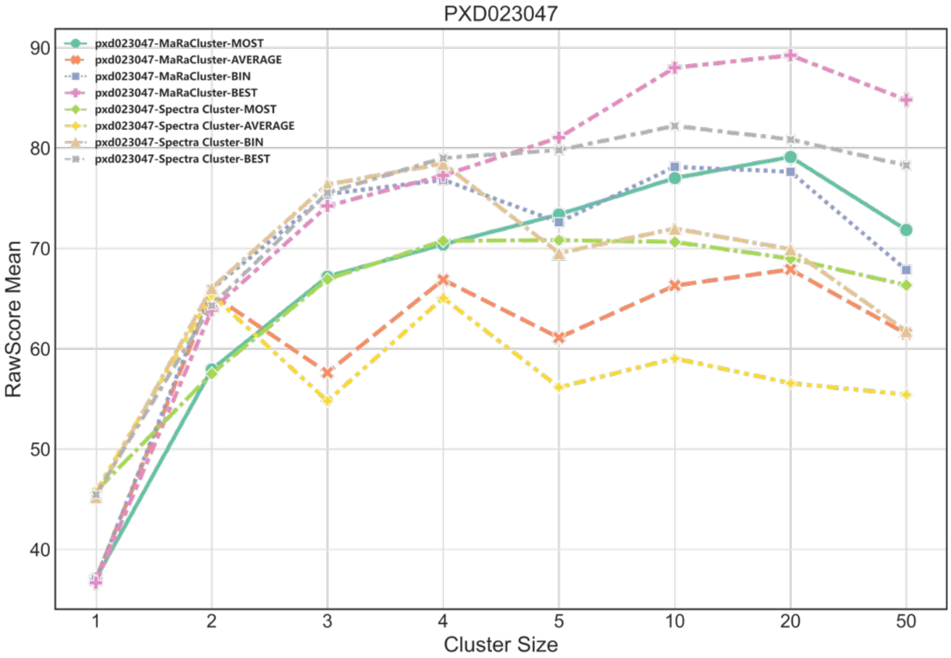

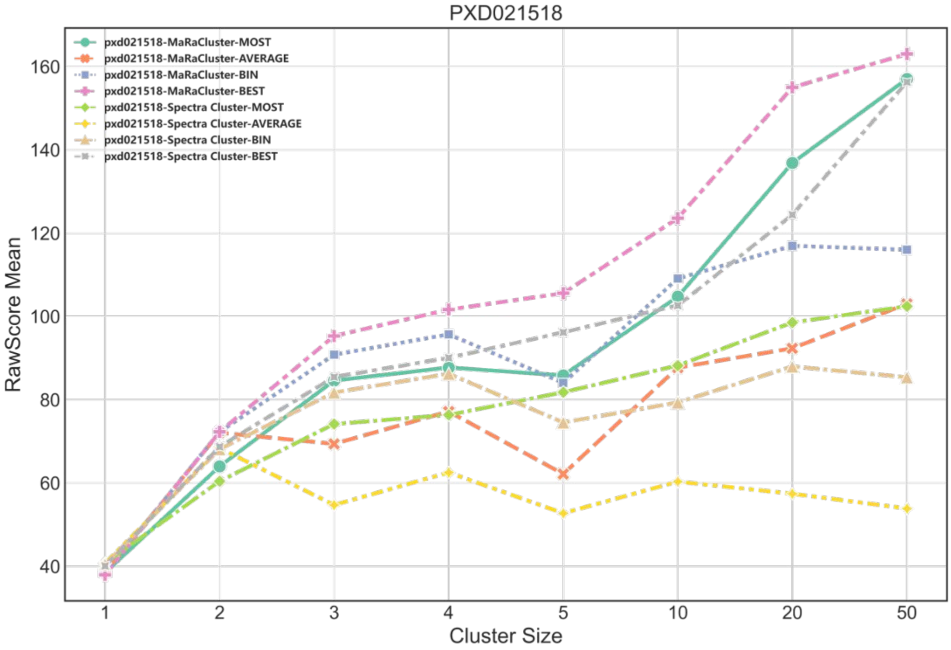

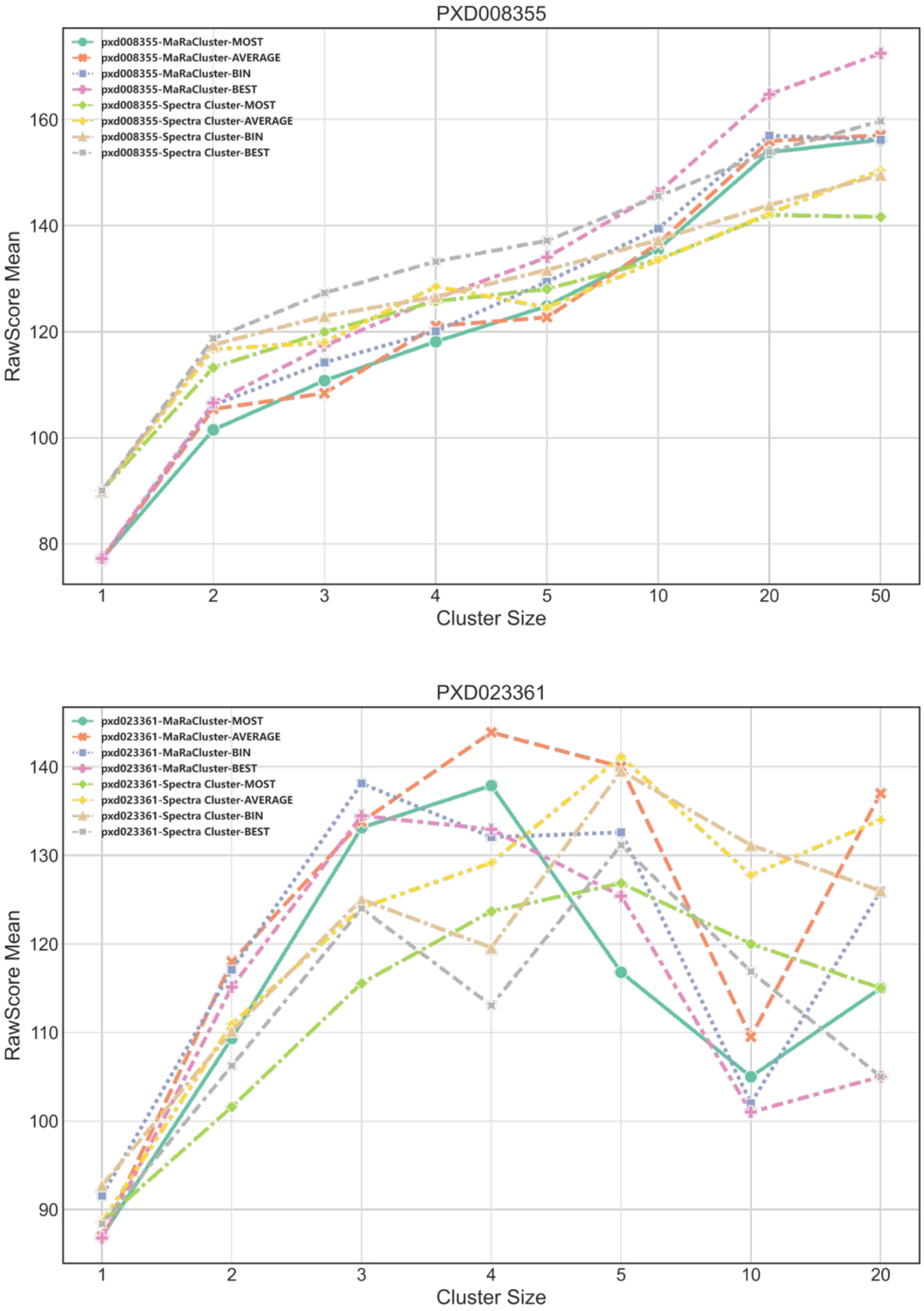

Figure S1: The average RawScore as a function of cluster size for the public proteomics datasets PXD023047, PXD021528, PXD008355, and PXD023361.

Supplementary Note 2: The benchmark datasets

PXD008355

MS name	No. MS	No. Unique Peptide	No. Unique Peptide (FDR=1)	No. PSMs	No. PSMs (FDR=1)	composite cluster RawScore mean	composite cluster Evalue mean
Original MS	1477567	278562	18153	772177	218794	-	-
MARACluster BEST	945661	252903	17283	540966	117368	139.77	0.00053
MARACluster BIN	1126561	250035	17355	539077	118506	133.28	0.00101
MARACluster MOST	1126561	253212	17282	544324	117685	128.62	0.00115
MARACluster AVERAGE	1126561	252316	17310	544667	118069	131.03	0.00109
spectra-cluster BEST	914502	248435	17181	522299	110737	144.73	0.00053
spectra-cluster BIN	1092343	246137	17225	521384	111722	134.00	0.00107
spectra-cluster MOST	1093792	248677	17183	525685	111305	129.38	0.00115
spectra-cluster AVERAGE	1093792	247808	17202	525716	111515	131.08	0.00113

PXD023047

MS name	No. MS	No. Unique Peptide	No. Unique Peptide (FDR=1)	No. PSMs	No. PSMs (FDR=1)	composite cluster RawScore mean	composite cluster Evalue mean
Original MS	109333	23649	8636	83138	52617	-	-
MARACluster BEST	56921	21032	8053	43502	21886	73.33	0.00400
MARACluster BIN	63306	21114	7904	43565	21424	72.05	0.00394
MARACluster MOST	63306	21119	7836	43442	21095	66.25	0.00400
MARACluster AVERAGE	63306	21068	7856	43470	21221	63.60	0.00397
spectra-cluster BEST	46993	19915	7941	35362	17950	73.99	0.00361
spectra-cluster BIN	52262	19878	7824	35135	17633	71.22	0.00405
spectra-cluster MOST	52653	20018	7672	35226	17159	65.46	0.00386
spectra-cluster AVERAGE	52653	20001	7710	35308	17352	60.63	0.00389

PXD021518

MS name	No. MS	No. Unique Peptide	No. Unique Peptide (FDR=1)	No. PSMs	No. PSMs (FDR=1)	composite cluster RawScore mean	composite cluster Evalue mean
Original MS	286410	104773	16427	172466	34173	-	-
MARACluster BEST	245917	102201	16073	157576	27290	79.54	0.00275
MARACluster BIN	265028	102170	15617	157429	26402	77.85	0.00263
MARACluster MOST	265028	102183	15575	157443	26320	70.48	0.00275
MARACluster AVERAGE	265028	102168	15605	157435	26381	71.93	0.00262
spectra-cluster BEST	241472	101782	15752	154257	25259	76.55	0.00272
spectra-cluster BIN	259656	101620	15363	153957	24532	73.29	0.00279
spectra-cluster MOST	259957	101666	15090	154030	24045	66.77	0.00271
spectra-cluster AVERAGE	259957	101630	15203	153984	24237	63.80	0.00262

PXD023361

MS name	No. MS	No. Unique Peptide	No. Unique Peptide (FDR=1)	No. PSMs	No. PSMs (FDR=1)	composite cluster RawScore mean	composite cluster Evalue mean
Original MS	38286	16109	1016	20223	2588	-	-
MARACluster BEST	34398	15359	1002	18606	2167	66.64	0.00282
MARACluster BIN	36520	15836	996	19142	2168	70.71	0.00240
MARACluster MOST	36520	15773	997	19134	2164	66.61	0.00290
MARACluster AVERAGE	36520	15806	998	19162	2164	66.66	0.00269
spectra-cluster BEST	33696	15225	1001	18177	2034	66.52	0.00288
spectra-cluster BIN	35707	15663	1003	18678	2054	70.54	0.00268

spectra-cluster MOST	35721	15641	998	18708	2035	66.68	0.00293
spectra-cluster AVERAGE	35721	15664	998	18726	2039	66.83	0.00273

Supplementary Note 3: Analysis of the phosphoproteomics dataset.

Cluster methods	Methods	No. PSMs with phosphory lation sites	No. phosphory lation sites	No. PSMs with identical sites	No. PSMs with different sites	No. PSMs with different peptides	No. unique PSMs
MaRaCluster	BEST	66914	81238	63165	2683	57	1009
	BIN	68429	83091				2524
	BEST	66914	81238	62979	1988	25	1922
	MOST	66555	80685				1563
	BEST	66914	81238	62872	2626	39	1377
	AVERAGE	67465	81924				1928
	BIN	68429	83091	63180	2436	27	2786
	MOST	66555	80685				912
	BIN	68429	83091	64576	2177	20	1656
	AVERAGE	67465	81924				692
	MOST	66555	80685	62958	2307	23	1267
	AVERAGE	67465	81924				2177
spectra-cluster	BEST	91195	109877	89161	1494	26	514
	BIN	92202	111230				1521
	BEST	91195	109877	89145	1145	20	885
	MOST	91259	109961				949
	BEST	91195	109877	89038	1475	24	658
	AVERAGE	91729	110648				1192
	BIN	92202	111230	89422	1343	16	1421
	MOST	91259	109961				478
	BIN	92202	111230	90180	1164	12	846
	AVERAGE	91729	110648				373
	MOST	91259	109961	89222	1340	15	682
	AVERAGE	91729	110648				1152

The parameters used by spectra-cluster were: Precursor tolerance (parameter: -precursor_tolerance) was set to 10 and the precursor tolerance unit (parameter: -precursor_tolerance_unit) was set to "ppm".

The fragment tolerance (parameter: -fragment_tolerance) was set to 0.5. The threshold starts (parameter: -threshold_start) was set to 1, and the end(parameter: -threshold_end) was set to 0.99995. We also specified -filter=mz_150 to remove all peaks below 150 m/. This is generally a good idea since these peaks do not contain amino acid fragment information. The rest of the parameters were left in their default settings.

We used MaRaCluster's default parameters. After clustering, the program created 5 files called MaRaCluster.clusters_p<x>.tsv (respectively p10, p15, p20, p25, p30) in a subdirectory called maracluster_output, for a range of p-value thresholds 10e-<x >. These output files contain one spectrum per line, with different clusters separated by an empty line. The spectrum is listed with the path to the spectrum file in the first column, the unmerged scannr (or scan index if no scannr is available) in the second column, and the clustered index in the third column, all separated by tabs. This experiment uses MaRaCluster.clusters_p30.tsv as the final result.
